# A multi-paradigm framework to assess the impacts of climate change on end-use energy demand

**DOI:** 10.1371/journal.pone.0188033

**Published:** 2017-11-20

**Authors:** Roshanak Nateghi, Sayanti Mukherjee

**Affiliations:** 1 School of Industrial Engineering and Division of Environmental and Ecological Engineering, Purdue University, West Lafayette, IN, United States of America; 2 Lyles School of Civil Engineering and School of Industrial Engineering, Purdue University, West Lafayette, IN, United States of America; Chongqing University, CHINA

## Abstract

Projecting the long-term trends in energy demand is an increasingly complex endeavor due to the uncertain emerging changes in factors such as climate and policy. The existing energy-economy paradigms used to characterize the long-term trends in the energy sector do not adequately account for climate variability and change. In this paper, we propose a multi-paradigm framework for estimating the climate sensitivity of end-use energy demand that can easily be integrated with the existing energy-economy models. To illustrate the applicability of our proposed framework, we used the energy demand and climate data in the state of Indiana to train a Bayesian predictive model. We then leveraged the end-use demand trends as well as downscaled future climate scenarios to generate probabilistic estimates of the future end-use demand for space cooling, space heating and water heating, at the individual household and building level, in the residential and commercial sectors. Our results indicated that the residential load is much more sensitive to climate variability and change than the commercial load. Moreover, since the largest fraction of the residential energy demand in Indiana is attributed to heating, future warming scenarios could lead to reduced end-use demand due to lower space heating and water heating needs. In the commercial sector, the overall energy demand is expected to increase under the future warming scenarios. This is because the increased cooling load during hotter summer months will likely outpace the reduced heating load during the more temperate winter months.

## Introduction

The U.S. energy infrastructure is capital intensive and requires significant investments in the planning and operation of the systems to ensure supply adequacy under a range of future contingencies. The ability to accurately predict and project future energy demand lies at the heart of sustainable and resilient planning and operation of the energy sector. This is because demand predictions drive supply expansion planning. It is to be noted that in this paper, we use the term ‘prediction’ to refer to short–and medium–term forecasts that rely on historical data, and ‘projection’ for longer–term forecasts that are contingent on scenarios used in the model. Inaccurate estimation of demand renders over- or under-investment in capacity expansion planning, which could lead to either rapid depletion of natural resources or large-scale supply shortages [1]. Energy demand forecasts are generally classified based on their lead-time windows of: (i) short-term (hourly, daily, weekly or monthly), (ii) medium-term (monthly to 1 to 5 year(s)), and (iii) long-term (5 to over 30 years of lead time). While short-term forecasts are key for reliable daily operations and medium-term forecasts are critical for supporting regulatory decisions related to rate-setting, long-term projections are important for identifying optimal investment strategies in capacity expansion decisions [2,3]. Each category of short, medium, and long-term forecast has its own unique challenges in terms of data availability, methodology, computational resources, types of stakeholders involved, and regulatory requirements. However, long-term energy projection is most complex due to the epistemic uncertainties about the future shifts in policy, technology, socio-economic conditions and climatic change. There exist many sophisticated energy-economy models for projecting medium- and long-term trends in the energy infrastructure such as MARKAL (MARKet ALlocation) [4], TIMES (The Integrated MARKAL-EFOM System) [5] and NEMS (National Energy Modeling System) [6]. While these models can account for factors such as the future changes in technology, socio-economic conditions, and policy impacts, they are not able to adequately incorporate uncertainties associated with shifts in end-use energy demand due to climate variability and change [3,7].

In this paper, we propose a data-driven, multi-paradigm methodology to characterize the complex energy demand–climate nexus in the residential and commercial sectors for various end-uses such as space cooling, space heating and water heating. Our proposed framework can be easily integrated with the existing energy-economy models (e.g., MARKAL) to account for climate sensitivity of the long-term end-use demands. While we selected the state of Indiana as a case study, our proposed approach can be readily extended to any other region in the United States. We selected Indiana due to a number of reasons detailed below:

1. Indiana is the 9^th^ most energy intensive state (on a per capita basis) in the U.S. [8];
2. Being an importer of electricity from the neighboring states and Canada, Indiana is vulnerable to supply shortages in cases of shifts in demand due to reasons such as climate variability and change [9];
3. The state of Indiana ranks 2^nd^ in terms of coal consumption in the energy sector. Deviated patterns of energy consumption, therefore, will have significant implications for the environment;
4. Indiana experiences varied states of climate such as lake-effect snows and winds off the Lake Michigan and localized weather variations in the hilly regions of the south. In general, the state experiences the interplay of polar air moving south from Canada and warm, moist air moving north from the Gulf of Mexico, which cause sudden variations in the climatic conditions, leading to complex patterns of energy demand for space conditioning [10].

Previous research has shown that unlike the transportation and industrial sectors, the residential and commercial sectors are most sensitive to climatic variability and change [3,11–14]. We therefore limited the scope of our analyses to the residential and commercial sectors.

## Background

Energy consumptions in the residential and commercial sectors are driven by complex interactions between socio-economic conditions, available technologies, land-use patterns, characteristics of the built environment, policy landscapes and climatic conditions of a given region. There exists a large body of literature on modeling energy demand in the residential and commercial sectors as a function of a wide range of factors such as climate, policy, technology, and socio-economic conditions [15–17]. However, since the objective of this paper is assessing the climate sensitivity of end-use demand, we focused our literature review on the studies that included climate variability and change as a key parameter in analyzing residential and commercial demand patterns.

### The residential sector

Steemers and Yun [18] developed a Generalized Linear Model (GLM)—using the cross-sectional Residential Energy Consumption Surveys (RECS) data in 2001—to examine the interactions between occupants’ behavior, building systems and climatic characteristics. Their objective was to examine the roles of occupant behavior (particularly in terms of space conditioning) and socio-economic factors in shaping energy demand curves. They fitted separate models for space heating and space cooling. Their results suggested that heating-degree-days (HDD) was the most significant predictor for space heating. In the case of space cooling, behavioral variables (e.g., extent of air-conditioning) were identified to be important. They also concluded that physical characteristics of buildings influenced the residents’ heating load more than their cooling load. Yun and Steemers analyzed the residential cooling load [19]. They assessed the relative significance of behavioral, physical and socio-economic parameters on cooling demand in order to provide a better understanding of their complex interactions, and to enable a more informed appraisal of interventions or incentives to improve energy efficiency. A few studies analyzed the role of ownership versus renting in shaping the climate-sensitive end-use demand. For instance, Levinson and Neimann [20] conducted a study to understand the energy consumption behavior of tenants residing in utility-included apartments by analyzing the EIA RECS data and the American Housing Survey (AHS) data (1985–1997). They hypothesized that tenants in utility-included apartments were less incentivized to conserve energy. Indeed, they found that tenants in utility-included apartments were inclined to set their thermostats 1–3°F higher during winters when they are away; contributing to 0.75% increase in fuel expenditures. The authors concluded that landlords were inclined to favor utility-included rent contracts. This is because, the property-owners could invest in energy efficient appliances (to reduce demand curves) while charging higher rents in exchange for paying utility bills upfront. Davis [21] leveraged the RECS 2005 dataset in order to investigate if renters were less likely to have energy efficient appliances. The study found significant variation in household energy use across different population groups, mainly due to economic factors and household characteristics. Min [22] developed a linear regression model using RECS 2005 data and U.S. Census Bureau 2000 zip-code-level data to predict energy used for space heating, water heating, cooling and appliances. Cities and suburban areas were found to have higher natural gas consumption compared to rural areas. They concluded that the total energy use was dominated by heating, with California ranking the lowest and some states in the Midwest and Northeast ranking the highest in heating energy demand. Kaza [23] trained a quantile regression model to the EIA RECS (2005) data to examine the relationship between energy consumption and various factors such as, heating and cooling degree days, total heating and cooling area, household size, price of electricity, housing type and age, neighborhood density, ownership and income. The study identified the age of the house as a more important predictor for heating rather than cooling. Total area was found to be positively related to household energy use, while neighborhood density did not seem to have a significant impact on energy demand. Moreover, cooling load was found to be inelastic with respect to the price of electricity. Howard [24] developed a multiple regression model to estimate demand intensity (Kilowatt-hour per square-meters of floor area, i.e., KWh/m^2^) in the built environment in New York City. They developed eight different models for various building types, but did not provide much information about the predictive performance of their models.

### The commercial sector

Yalcintas [25] developed energy benchmarking models—based on the Artificial Neural Networks (ANN)—for office buildings in all census regions of the U.S. The ANN models outperformed linear regression in terms of both goodness-of-fit and predictive accuracy. Their analysis focused on model's predictive performance and no statistical inferences were discussed in their paper. The Department of Energy also conducted a benchmarking study based on energy usage intensity (EUI) to make recommendations based on principal building activity [26]. Similarly, the Pacific Northwest National Laboratories published recommendations based on (non-statistical) inferences from the Commercial Building Energy Consumption Surveys (CBECS 2003) dataset to suggest HVAC systems for buildings based on their principal activity [27]. Another study [28] assessed the feasibility of achieving net zero-emissions in commercial buildings. The authors discussed the concept of Zero Energy Building (ZEB) potential, using the ‘EnergyPlus’ simulation package. Their results indicated that 22%–64% of buildings could potentially reach ZEB by 2005–2025.

Derrible and Reeder [29] used the Department of Energy's eQuest v3.65 package to simulate the energy consumption in the U.S. commercial buildings. They concluded that the U.S. commercial buildings are 'over-cooled' and quantified the associated economic costs. Blum and Sathye [30] analyzed the non-governmental, and non-mall commercial buildings in the U.S. to examine whether a market failure due to principal-agent (PA) problem might have prevented the installation of energy-efficient devices, or efficient operation of space-conditioning equipment. The study identified PA market failures for smaller buildings (i.e., area less than 50,000 sq. ft.) in the case of space heating and market failures for all building types in the case of space cooling.

### Existing gaps and our contribution

While reviewing the significant developments in the area of energy demand–climate nexus (outlined above), we have identified a number of knowledge gaps in the field, some of which are summarized below.

1. Majority of the statistics-based approaches used for modeling the residential sector’s demand, leverage linear models to characterize the energy demand–climate nexus. However, recent research findings have shown that linear models are inadequate in capturing the complex and non-linear relationship between energy demand and climate variability and change [3,11,16]. On the other hand, the non-linear methods used for modeling an individual home’s demand trends (e.g., [31]) cannot be easily rolled up to represent the entire residential energy sector).
2. Most of the existing studies have focused on characterizing the end-use demand sensitivity to climate variability, using *cross-sectional* data. While analyses based on cross-sectional data can help to extract useful information for a given point in time, they are not well-suited for medium- and long-term projections. This is because, cross-sectional data lack information on temporal variability. It should be noted that other studies have leveraged time-series data to capture medium- and long-term sensitivity of energy demand to climate [3,11–14,16, 32]. However, such studies used aggregate energy consumption data, with no breakdown of the climate sensitive portion of the end-use demand such as space heating, space cooling and water heating. Therefore, the results of such models (e.g., [3,11–14,16]) cannot be integrated into energy-economy models (e.g., MARKAL and NEMS).

To address these knowledge gaps, we developed a multi-paradigm framework to characterize the climate sensitivity of end-use demand. More specifically, we developed a Bayesian, non-parametric data-miner to characterize the state-level energy demand–climate nexus in the residential and commercial sectors. The aggregate demand estimates were then mapped onto statistically representative individual user units (i.e., house-hold level in the residential sector, and building level in the commercial sector). Downscaled climate change scenarios were then used to project future end-use demand for space cooling, space heating and water heating at the individual household and building levels. Our model outputs can easily be integrated into energy-economy models such as MARKAL to enable accounting for climate variability and change while projecting the medium- and long-term energy demand under various policy scenarios.

## Data collection and visualization

In our analysis, we included four categories of data, viz., (i) state-level, time-series data of energy demand in the residential and commercial sectors, (ii) fractions of total end-use energy needed for space cooling, heating and water heating for the residential households and commercial buildings, (iii) historical climate data for the state of Indiana, and (iv) statistically downscaled projections of future climate data for the state. Below, we will briefly discuss each of these categories of data.

The state-level annual net end-use energy demand data for the residential and commercial sectors were obtained from the U.S. EIA State Energy Data System (SEDS) for the period of 1963–2014 [33]. This yearly sectoral energy demand accounts for only the net energy consumption, excluding the electrical system energy losses. More specifically, the net energy demand represents aggregated consumption of coal, petroleum, natural gas, electricity (including energy losses during transmission), biomass, geothermal and solar energy use in a year and is expressed in trillion Btu (British thermal units) [33]. It is noteworthy that we only included data post 1980’s to exclude the tipping point effects of the oil and energy crises in the 70’s [34,35]. The fractions of different categories of end-use energy (i.e., space heating, space-cooling and water heating) were obtained from the EIA Residential Energy Consumption Survey (RECS)-2009 and Commercial Buildings Energy Consumption Survey (CBECS) datasets-2012 [36,37].

Table 1 provides the residential sector’s descriptive statistics on (i) historical net energy consumption during 1981–2013 from the SEDS dataset, and (ii) the fractions of climate sensitive end-use energy demands (i.e., space heating and cooling and water heating) extracted from the most recent version of the EIA RECS (2009) dataset [36]. EIA RECS data contains granular information on a ‘statistically representative’ sample of households in the states of Indiana and Ohio. In the RECS dataset, states with lower population levels and similar energy consumption profiles are combined and therefore data exclusive to the state of Indiana is not available. However, since the states of Indiana and Ohio are quite similar in terms of their demographics and energy profiles, the implications of using such data in our analysis will likely be minimal.

**Table 1:** Descriptive statistics of end-use energy consumption (in trillion Btu) in the residential sector.

Variable	Min.	1^st^ Q.	Median	Mean	3^rd^ Q.	Max.
Net energy consumption (1981–2013)	256.6	276.1	288.7	286.3	295.6	315.0
Space cooling fraction	0.0	0.003	0.009	0.017	0.017	1.000
Space heating fraction	0.0	0.690	0.745	0.731	0.820	0.970
Water heating fraction	0.0	0.172	0.245	0.252	0.294	0.989

From the Table 1 above, we can observe that the greatest fraction of the energy demand in Indiana is attributed to space heating and water heating. Space cooling accounts for a very small fraction of the total statewide energy consumption. More specifically, on average, 73.1% of total energy is consumed for space heating, 25.2% is used for water heating and about 1.7% is needed for space cooling.

Table 2 provides the commercial sector’s descriptive statistics on (i) historical net energy consumption during 1981–2013 from the SEDS dataset, and (ii) fractions of climate sensitive end-use energy demand obtained from a statistically representative sample of commercial buildings in the East North Central region available at EIA CBECS-2012 [37]. Unfortunately, granular data at individual state-level is not available. The assumption here is that the commercial end-use energy profile for Indiana is similar to that of the entire East North Central region of the U.S.

**Table 2:** Descriptive statistics of end-use energy consumption (in trillion Btu) in the commercial sector.

Variable	Min.	1^st^ Q.	Median	Mean	3^rd^ Q.	Max.
Net energy consumption (1981–2013)	123.7	155.5	171.2	168.1	183.1	198.5
Space cooling fraction	0.0	0.060	0.126	0.157	0.222	0.797
Space heating fraction	0.0	0.007	0.051	0.151	0.234	0.980
Water heating fraction	0.0	0.002	0.007	0.052	0.048	0.844

Unlike the residential sector, the fraction of total commercial energy demand attributed to the space cooling is comparatively much higher (Table 2). Space cooling accounts for 15.7% on average of the total statewide energy consumption while, space heating and water heating respectively consumes 15.1% and 5.2% of the total energy on average.

**Fig 1:**
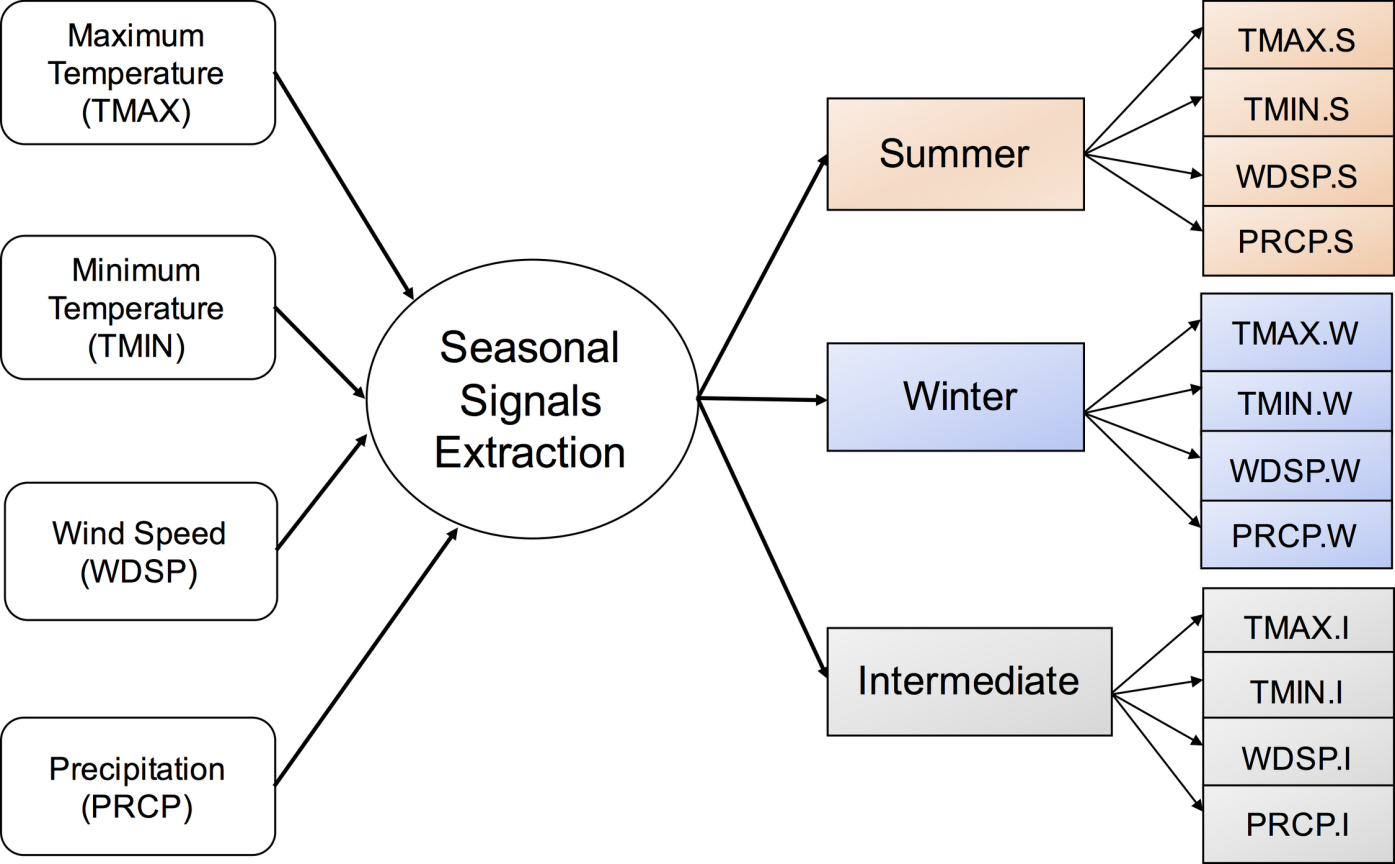
Classification of input climate variables.

Global monthly estimates of the historical climate data for the state of Indiana was collected from the National Centers for Environmental Information (NCEI) of National Oceanic and Atmospheric Administration (NOAA) for the period of analysis [38]. Initially, data was collected from all climate stations in Indiana, and then filtered to include only the stations with complete information [3]. To extract the seasonal signals in climate data, we followed a three-step procedure: (i) aggregated the station-averaged daily data to monthly-level data using the monthly medians; (ii) classified the weather variables into three seasonal categories of summer (June–September), winter (December–March) and intermediate (April, May, October, November), to be consistent with the seasonal classifications in the commonly used EIA energy-economy models, such as MARKAL (Fig 1), and (iii) computed the seasonal means and standard deviations for each of the climate variables.

In our previous research, we identified dew point temperature, average wind speed, and precipitation levels as the key predictors of the climate sensitive portion of the end-use demand [11, 16]. However, since projections of downscaled dew-point temperature were unavailable, we used minimum and maximum temperature as proxies for the dew point temperature. This is reasonable as minimum temperature (TMIN) represents the lower range of the dew point temperature, while the maximum temperature (TMAX) captures the higher range of the dew point temperature [18].

**Fig 2:**
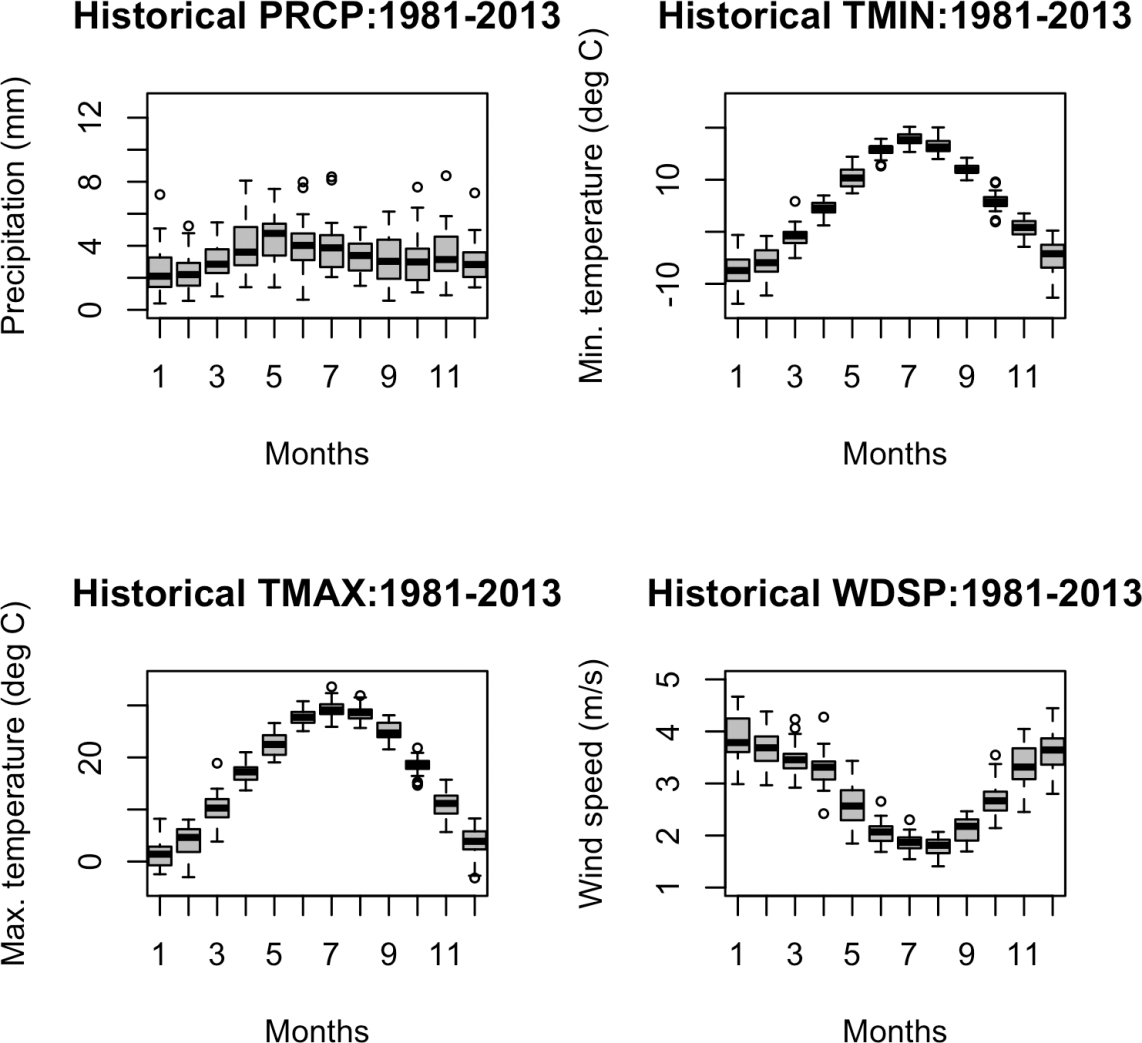
Historic patterns of climate predictors, namely (a) total precipitation, (b) minimum temperature, (c) maximum temperature and (d) wind speed.

The seasonal distribution of the historical climate data is given in Fig 2. We observe that precipitation levels tend to be highest during spring, but does not vary significantly seasonally as compared to the temperature and wind speeds. Wind speed is observed to be lowest during the summer months and reaches peak during the winter months.

### Climatic data projections

**Fig 3:**
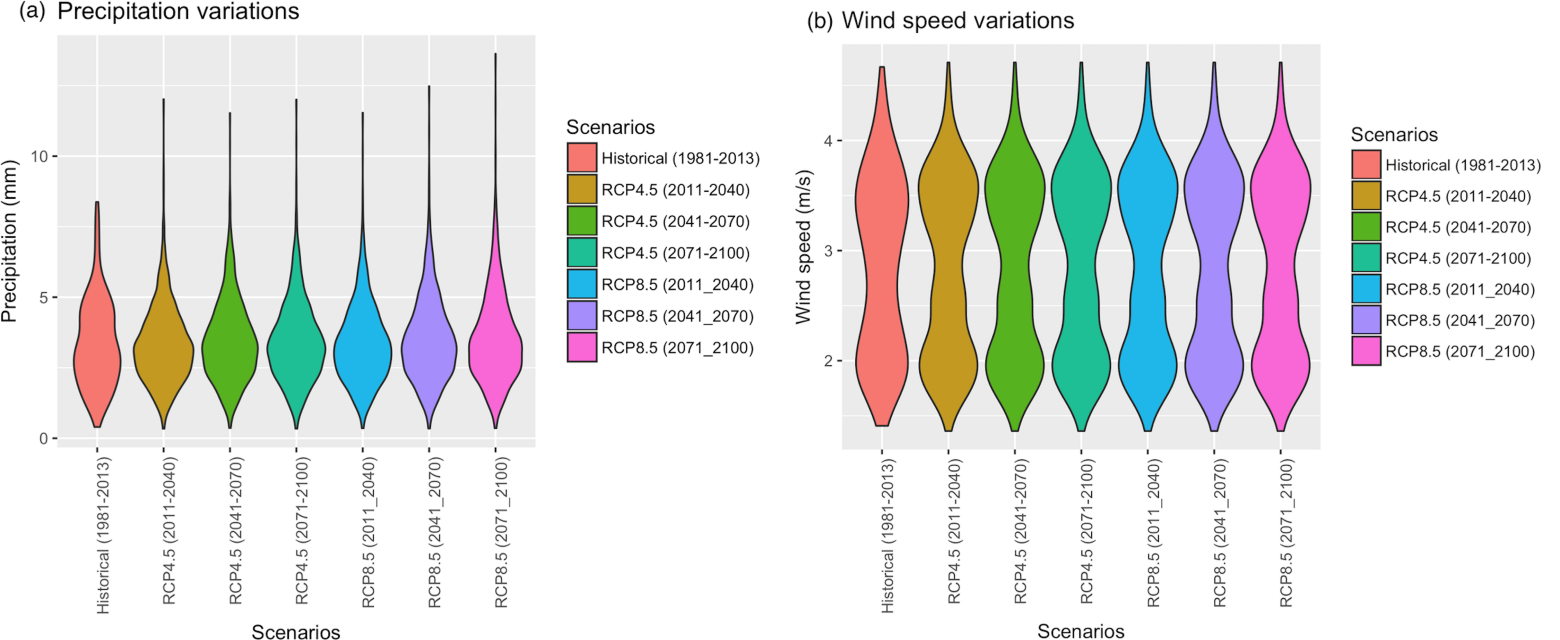
Scenario-based comparisons of (a) precipitation data, and (b) wind speed data.

**Fig 4:**
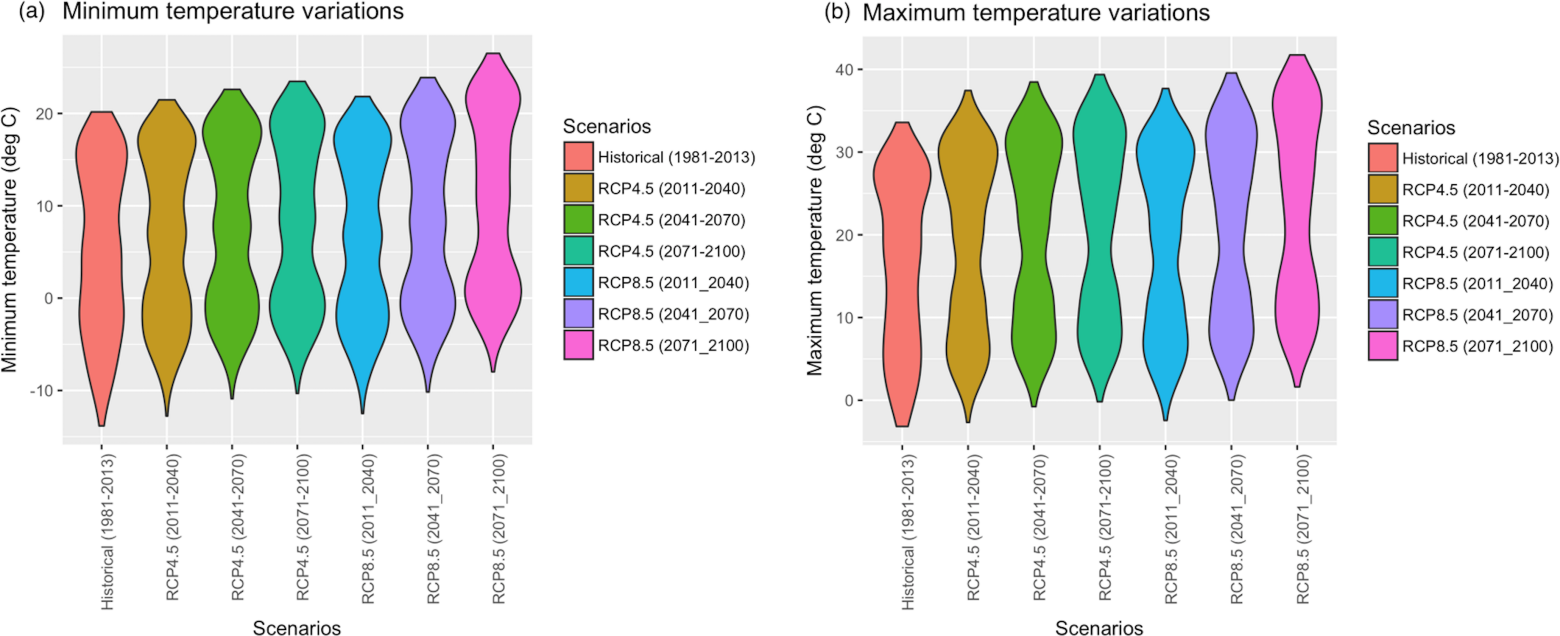
Scenario-based comparisons of (a) minimum temperature, and (b) maximum temperature data.

To assess the long-term climate sensitivity of the residential and commercial end-use demand in Indiana, we used the Representative Concentration Pathways (RCP), adopted by the IPCC 5^th^ Assessment Report [39,40]. RCPs, represent a large set of scenarios in the literature and characterize possible development trajectories for the key forcing agents of climate change [40]. More specifically, we used the two scenarios, namely, RCP 8.5 (characterized by high greenhouse concentration levels) and RCP 4.5 (representing a more stabilized scenario). Due to the coarse spatial resolution of the climate scenarios, they were downscaled to the State of Indiana using the hybrid-delta technique [41]. Figs 3 and 4 show the distribution of historical and projected input climate variables under the two scenarios. It can be seen from Fig 3A, that while there seems to be a modest upward trend in the average value of precipitation, the tail of its probability density function becomes much heavier under RCP 8.5; indicating an increased likelihood of experiencing extremes under the high emission scenario. Based on Fig 3B, there is little evidence that the distribution of wind speed will statistically change in the future.

It can be seen from Fig 4A and 4B respectively that both minimum and maximum temperatures are increasing over time, with larger shifts under RCP 8.5 relative to RCP 4.5.

## Methodology

**Fig 5:**
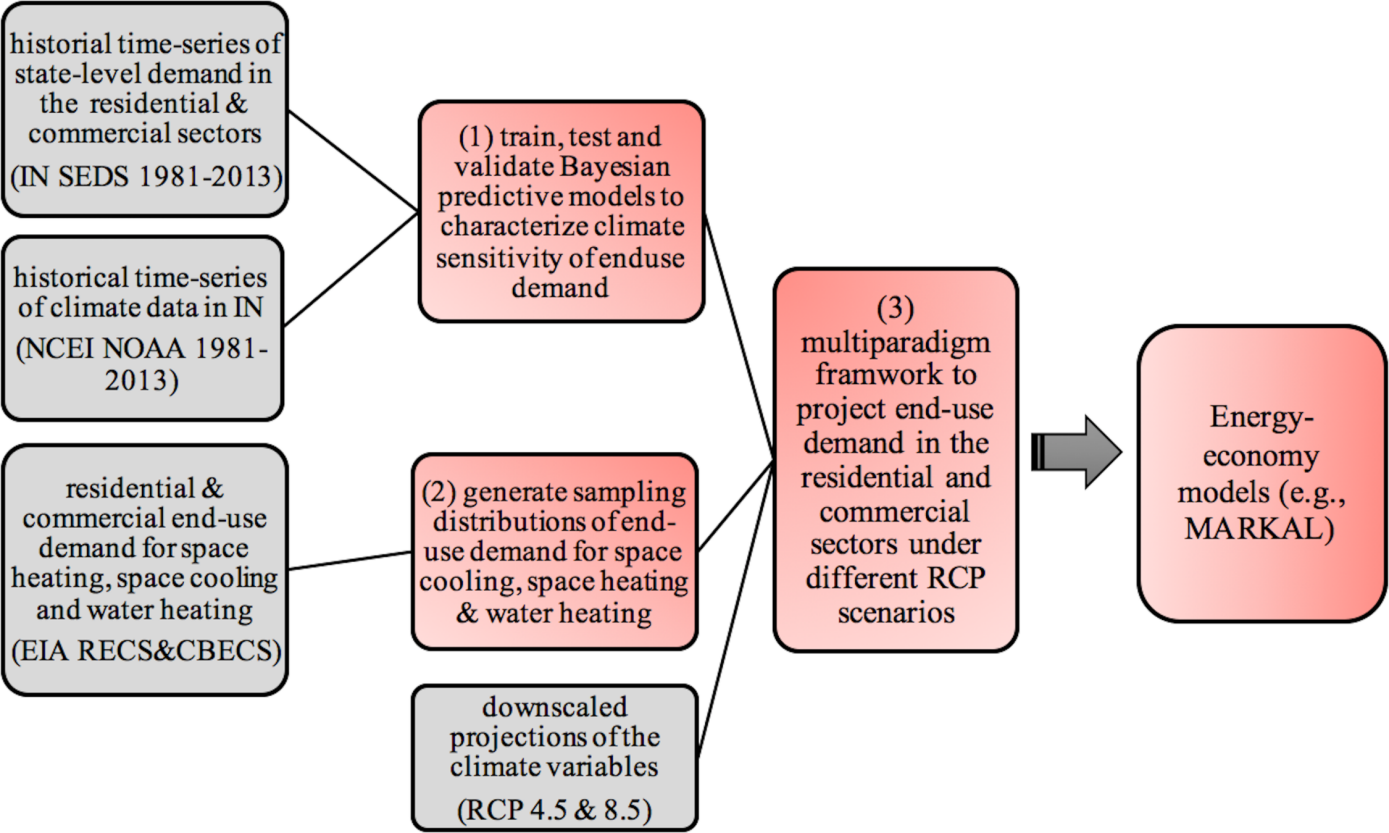
Schematic of our proposed approach. The gray boxes represent input data sources, and the red boxes represent model developments.

Fig 5 below summarizes our multi-paradigm framework for projecting the residential and commercial end-use energy demands into the future (until 2100) under the various climate change scenarios. We refer to our approach as a “multi-paradigm” framework because it integrates different types of modeling techniques, such as, simulation, machine learning and hybrid downscaling on the same platform to obtain our final outcomes.

The final estimates from our proposed framework can then be used as input to the energy-economy models (e.g., MARKAL) to model the long-term impacts of climate change on the regional energy demand under various future climate scenarios. The details of each of the steps in creating our proposed multi-paradigm framework (i.e., steps 1–3 in the red boxes in Fig 5) are outlined below.

### Train, test and validate Bayesian predictive models to characterize climate sensitivity of demand in the residential and commercial sectors: Step 1

State-level annual net energy demand in the residential and commercial sectors [33], together with the historical monthly climate data [38] were used to develop Bayesian predictive models for each sector. To harmonize the temporal scale of our climate data with the seasonal scale, typically used in most energy-economy models (such as MARKAL [4] and NEMS [6]), we aggregated the climate data over the three seasons of summer (June–September), winter (December–March) and intermediate (April, May, October, November). In training our predictive models, we included the variable “year” in addition to the (climatic) predictors (discussed in the data section) to control for the (non-climatic) secular trends.

Based on our previous research results [3, 11], we leveraged the method of Bayesian additive regression trees (BART) [42] to develop our predictive models. Our prior research found BART as a particularly powerful algorithm for modeling the energy demand–climate nexus due to its: 1) superior predictive performance, 2) ability to yield fully probabilistic prediction and credible interval uncertainty estimation, and 3) ability to incorporate expert knowledge into the learning procedure [3, 11]. We conducted a thorough cross-validation process in training, testing and validating our energy forecasting models. More specifically, we used a 20% randomized holdout analysis to estimate the predictive accuracy of the models and simulated the process for 30 times—as a conservative measure to ensure that all data has been used at least once. The expected values of MSE (Mean Square Error) and MAE (Mean Absolute Error) were used to estimate the out-of-sample prediction accuracy for the models developed for each sector.

#### Background theory on the approach used

Bayesian, additive regression-trees (BART) [42–44] is a sum-of-trees model where the response function is approximated by aggregating the estimates from *m* ‘small’ decision trees. Mathematically, BART can be represented as:

$$Y=[\sum_{j=1}^{m}g(X;T_{j},M_{j})]+\epsilon ,\;\mathrm{where}\;\epsilon ∼N(0,\sigma^{2})$$ (1)

In the Eq (1) above, the additive stochastic component *ϵ*–referred to as irreducible error—reflects the dependence of the response variable on quantities other than the input variables that are neither observed, nor measured. If a model accurately approximates the response variable, the irreducible error *ϵ* will be normally distributed with an expected value of zero. This assumption is usually checked by plotting normal quantile (QQ) plots of the model’s residuals. The function *g*(***X***; *T*_*j*_, *M*_*j*_) in the Eq (1) above, assigns the parameters ‘*M’* of tree *T* to the *p*-dimensional predictor ***X*** across all *m* trees. Specification of prior probabilities (on both tree structures and the conditional expectations at each terminal node) are used to (i) control model complexity and (ii) incorporate expert knowledge, for instance by expressing a preference for a certain attribute in the model. Combining the prior distributions with tree-model likelihoods yields a posterior distribution on the tree models [42]. Metropolis-Hastings algorithm is typically used to characterize the posterior probability space [42,43].

Since the algorithm used in developing our predictive models is non-parametric, variable ranking and partial dependence plots (PDP) were generated to facilitate model inference. The variable importance ranking was computed based on ‘variable inclusion proportion’ that indicates the fraction of times a given predictor was used in constructing a decision-tree. PDPs are generally used for conducting variable inference for non-parametric statistical models. They help establish the individual effects of the predictor variables *x*_*j*_ in a ceteris paribus condition (i.e., controlling for all the other predictors). Mathematically, the estimated PDP is given as shown in Eq (2) below:

fj^(xj)=1n∑i=1nfj^(xj,x−j,i) (2)

Here, $\hat{f}$ represents the statistical response surface; *n* stands for the number of observations in the training dataset; *x*_−*j*_ denotes all other variables (except for *x*_*j)*_. The estimated PDP of the predictor *x*_*j*_ indicates the average value of the estimated response function $\hat{f}$ when *x*_*j*_ is fixed and *x*_−*j*_ varies over its marginal distribution.

The generalization performance of any statistical model depends on its ability to produce accurate predictions on an independent test sample [43, 44]. Bias-variance trade-off is central for minimizing the generalization error [43]. Cross-validation is one of the most widely used resampling techniques for balancing bias-variance trade-off in statistical models [11, 43]. More specifically, *k*-fold cross-validation is commonly leveraged to assess statistical models’ predictive accuracy (aka generalization performance). *k*-fold cross-validation involves randomly subdividing the dataset into *k* (equally-sized) subsets. In each iteration, the statistical model is fitted to the training subset that includes all observations except for the *k*^*th*^ held-out sample. The predictive accuracy is then calculated based on the model’s performance on the *k*^*th*^ held-out subset. This process is repeated until all data is used at least once and the average performance across all iterations is recorded as a measure of the model’s predictive performance.

### Generate sampling distributions of end-use demand for the residential and commercial sectors: Step 2

The fractions of the *house-hold-level* energy used for space cooling, space heating and water heating were extracted from the RECS (2009) dataset [36]. Similarly, the fractions of *commercial building-level* energy used for space cooling, space heating and water heating were extracted from the CBECS (2012) dataset [37]. Since the empirical distributions of end-use demand for space heating, space cooling, and water heating were found to be heavy-tailed, we used the generalization of the central limit theorem [45, 46] to generate sampling distributions of the end-use demand proportions in each sector. To map the aggregate estimates of state-level demand onto the individual household-level/building-level estimates, we multiplied the aggregate demand by the generated probability density functions of the end-use demand proportions for space heating, space cooling and water heating. The implicit assumption here is that the distributions of these three types of end-use energy consumption will not significantly change over time. This assumption may be invalid in the face of major technological or behavioral shifts in the future. In the presence of strong evidence for certain structural shifts in technology or behavior, the generated sampling distributions of end-use demand can be updated—based on the empirical data or expert knowledge—to reflect such information.

### Project residential and commercial end-use demand under different climate scenarios: Step 3

To project the climate sensitive portion of end-use demand under a future climate scenario, the Bayesian predictive models for the residential and commercial sectors were trained separately with the downscaled climate data associated with that scenario. It should be noted that our Bayesian predictive models estimated the median values of end-use demand together with uncertainty intervals i.e., Bayesian credible intervals and prediction intervals. Bayesian credible interval characterizes the uncertainty, given the posterior distribution of the response variable [44] and the prediction interval characterizes uncertainties associated with future variables that are yet not observed. The annual projections for each sector were then multiplied by the generated empirical distributions of the end-use demand for space heating, space cooling and water heating (outlined in step 2 above). The probabilistic estimates of the end-use demand for space heating, space cooling and water heating for the residential and commercial sectors can be used as inputs in the most commonly used energy-economy models, to allow for accounting the impacts of climate variability and change under different scenarios.

## Results

The performance of our predictive models developed to characterize the energy demand–climate nexus for the residential and commercial sectors in Indiana are summarized in Tables 3 and 4. To benchmark the performance of our predictive models, we also provided information about the ‘null’ or ‘mean-only’ models. Comparison with the ‘null model’ reveals the extent of the predictive model’s contribution to explaining the variance of the response, beyond its historical mean [11]. It can be seen from Table 3 that the predictive model—based on the BART algorithm—for the residential sector offers a 26% improvement in out-of-sample RMSE, and 32% in terms of out-of-sample MAE compared to the “mean-only” alternative. Moreover, the in-sample improvements (in terms of RMSE and MAE) associated with the BART-model over the null-model are much more significant (Table 3).

**Table 3:** Predictive performance: The residential sector.

#	Model	R^2^	In-sample		Out-of-sample	
			RMSE	MAE	RMSE	MAE
1	Null Model (i.e., Mean-only model)	-NA-	14.45	12.10	15.19	12.99
2	BART Predictive Model	0.96	2.72	2.04	11.25	8.87

**Table 4:** Predictive performance: The commercial sector.

#	Model	R^2^	In-sample		Out-of-sample	
			RMSE	MAE	RMSE	MAE
1	Null Model (i.e., Mean-only model)	-NA-	19.14	15.72	9.15	18.60
2	BART Predictive Model	0.99	1.22	0.94	7.06	15.42

Similarly, it can be seen from Table 4, that the BART-predictive model for the commercial sector offers a significant improvement over the ‘mean-only’ model. More specifically, the BART model’s out-of-sample accuracy is 23% higher in terms of RMSE, and 17% in terms of MAE. Similar to the results for the residential sector, our predictive model for the commercial sector outperforms the null-model in terms of in-sample errors even by a greater margin.

**Fig 6:**
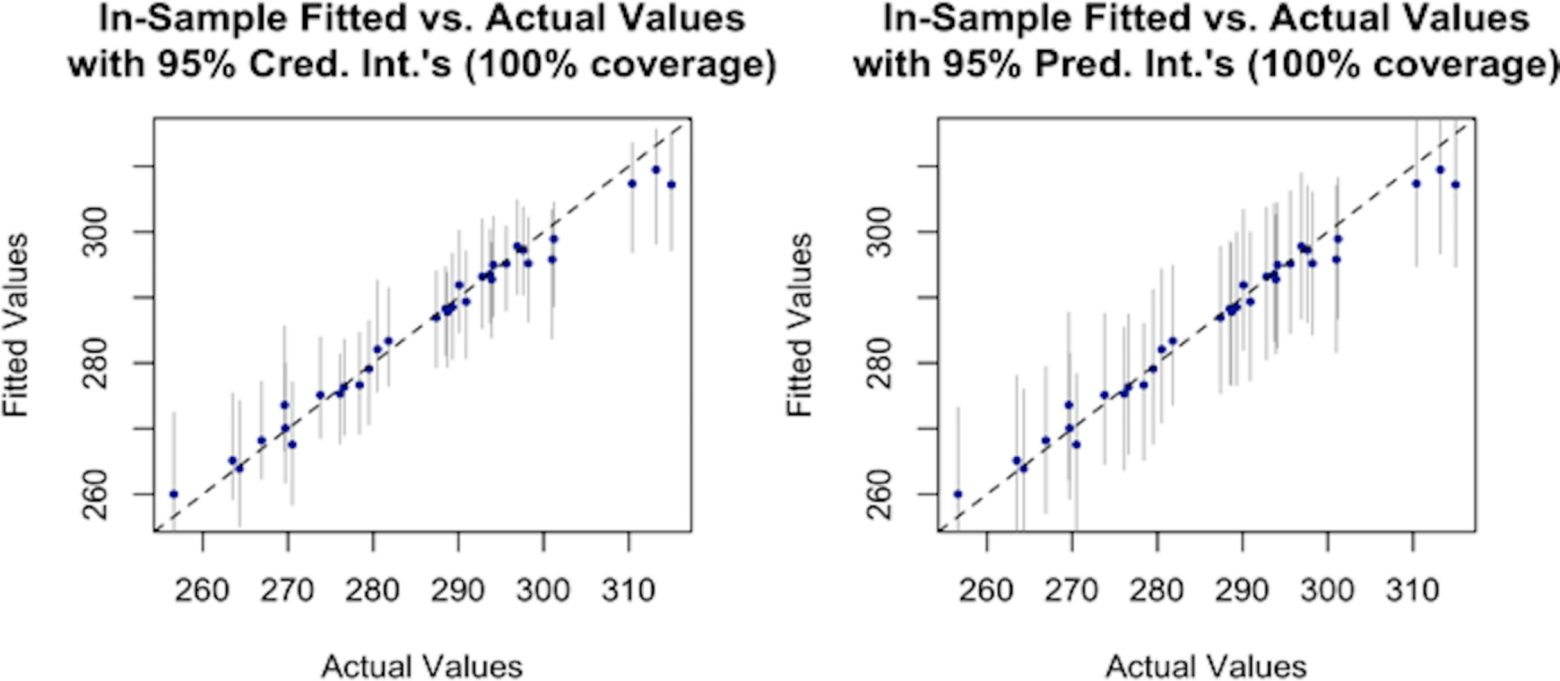
Plot of fitted versus observed values of total end-use consumption (trillion Btu) in the residential sector.

**Fig 7:**
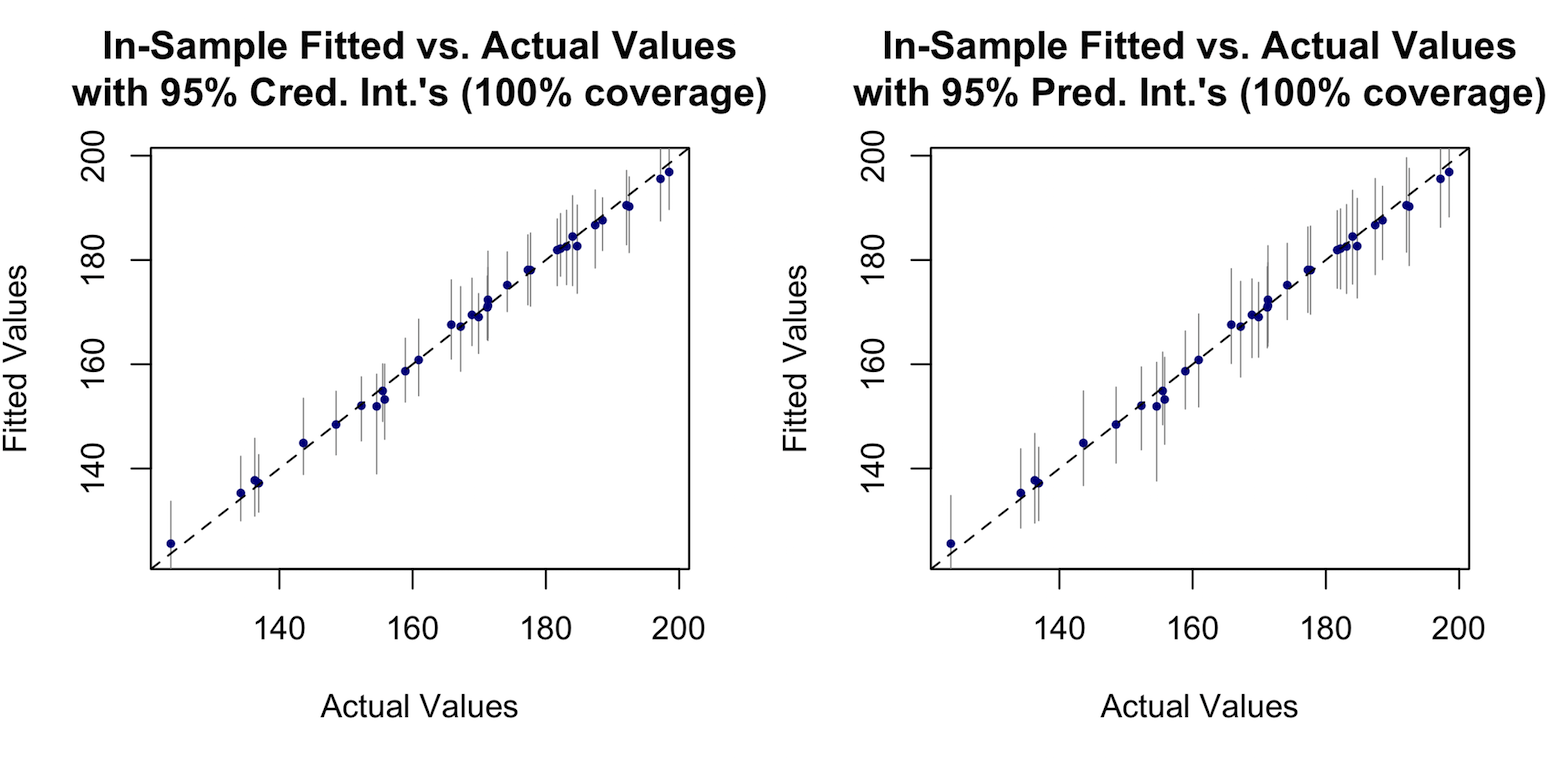
Plot of observed versus fitted values of total end-use consumption (trillion Btu) in the commercial sector.

It can be also seen from Tables 3 and 4 that the predictive models for both the sectors fit the data remarkably well, as evidenced by the substantially high R^2^ values of 96% for the residential sector and 99% for the commercial sector. The models’ goodness-of-fit, together with their uncertainty bounds are visualized for the residential and commercial sectors in Figs 6 and 7.

The plots of the estimated values versus observed values for both sectors include information about both the credible intervals, and prediction intervals. It can be observed from Figs 6 and 7, that the uncertainty bounds are wider in the residential sector, compared to the commercial sector.

**Fig 8:**
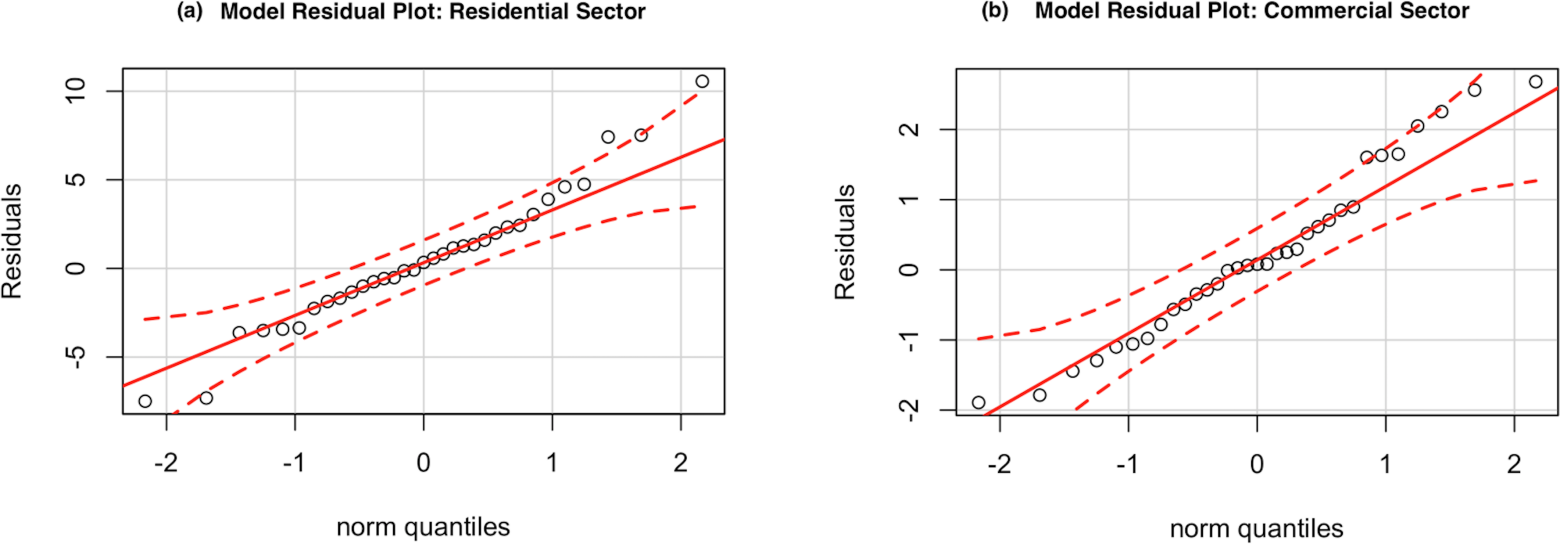
**Residual plots for (a) the residential sector and (b) commercial sector.** The red dashed lines represent the 95% confidence intervals.

Fig 8 depicts the normal quantile-quantile (Q-Q) plots of the residuals for (a) the residential, and (b) the commercial sectors. Since all the points lie along the 45° line of the normal quantile plots—and within the 95% confidence intervals (red dotted lines)—it can be concluded that the residuals are normally distributed. This indicates that the developed models for both sectors have adequately characterized the variability in the response variable. The normally distributed residuals, with an expected value of zero, represent the inherent stochasticity associated with the response variable that is independent of the input variables (refer to Eq (1) in the methodology section).

### Model inference

**Fig 9:**
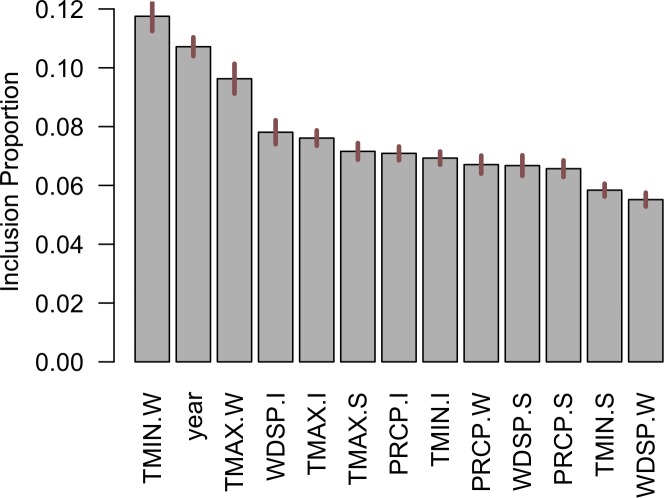
Ranking of variable importance in predicting end-use residential energy consumption.

**Fig 10:**
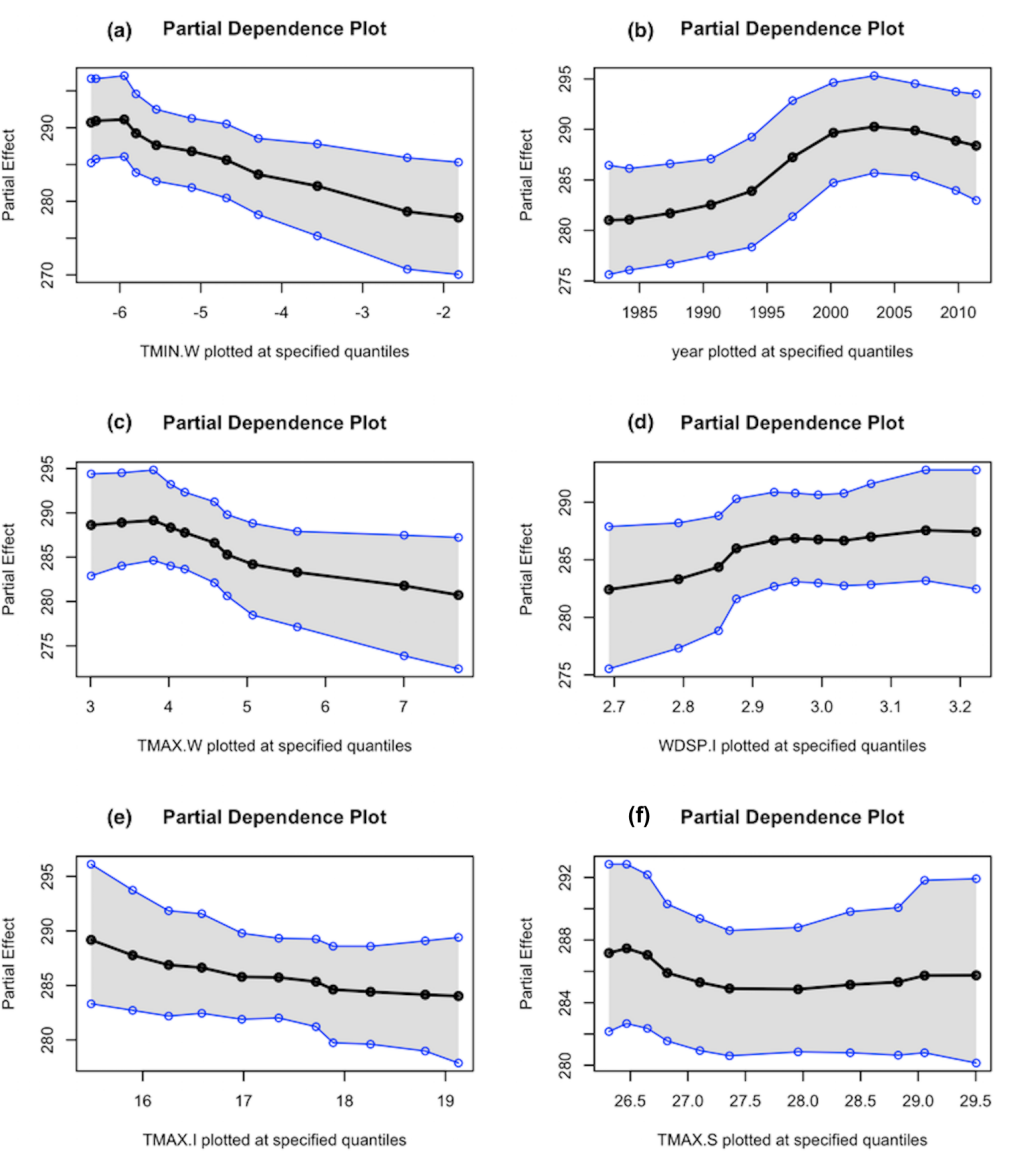
Partial dependencies of the top six key predictors of (a) minimum temperature (in °C) during the winter, (b) year, (c) maximum temperature the during winter (in °C), (d) wind speeds during the intermediate season (in m/s), (e) maximum temperature during the intermediate season (in °C) and (f) maximum temperature (in °C) during the summer, in the residential sector.

Since our predictive model is non-parametric, we leveraged variable importance bar-charts as well as partial dependence plots to make statistical inferences. Fig 9 shows the bar-chart of variable importance rankings for the residential sector. The variable ranking is based on ‘variable inclusion proportion’ which indicates the fraction of times a given predictor was used in constructing a decision-tree in the ensemble model. The bar-chart also includes uncertainty bounds, showing the variation in inclusion proportion across all decision trees used in the BART ensemble model. It can be observed that the temperature-related variables during the winter months (i.e., TMIN.W and TMAX.W) are ranked highest in terms of importance. This is expected, since the largest fraction of the energy consumption in the residential sector in Indiana is attributed to space heating (Table 1). The variable ‘year’ shows up as a key predictor which is expected because it captures the secular trends in energy consumption data. Maximum temperature during warmer months (summer and intermediate seasons), and wind speeds during the intermediate months (April-May and October-November) are also identified as important predictors. Precipitation patterns appear to rank lower than temperature and wind-related variables in the residential sector. To understand the relationship between each of these key climatic variables and energy demand, partial dependencies were plotted (Fig 10).

It can be seen from Fig 10A, 10C and 10E that the minimum and maximum temperatures during the winter (i.e., during December-March), and maximum temperature in intermediate months (i.e., April-May, and October-November) are negatively associated with energy demand. This is expected because higher temperatures during the colder months result in reduced demand for space heating. Fig 10B shows that the energy consumption in the residential sector in the state of Indiana increased steadily during 1980–2000. The consumption levels plateaued during the years 2000–2005, and then started to follow a downward trend after mid-2000’s. The downward trend since mid-2000’s can be attributed to several energy efficiency initiatives. such as, the ‘Energizing Indiana’ program that contained comprehensive energy-efficiency programs for Indiana home-owners.

Wind-speed during the intermediate season is positively associated with residential end-use demand. Referring to Fig 2, it shows that wind speeds during the fall (i.e., months of October and November) are comparatively stronger than the other seasons in the state of Indiana, which can cause cooling effects, thereby increasing demand for space heating and water heating. While maximum temperature during the summer is also an important predictor, its impact on the residential energy demand is comparatively attenuated. The attenuated effect is largely attributable to the fact that only a small fraction of energy is used for space cooling in the state of Indiana.

**Fig 11:**
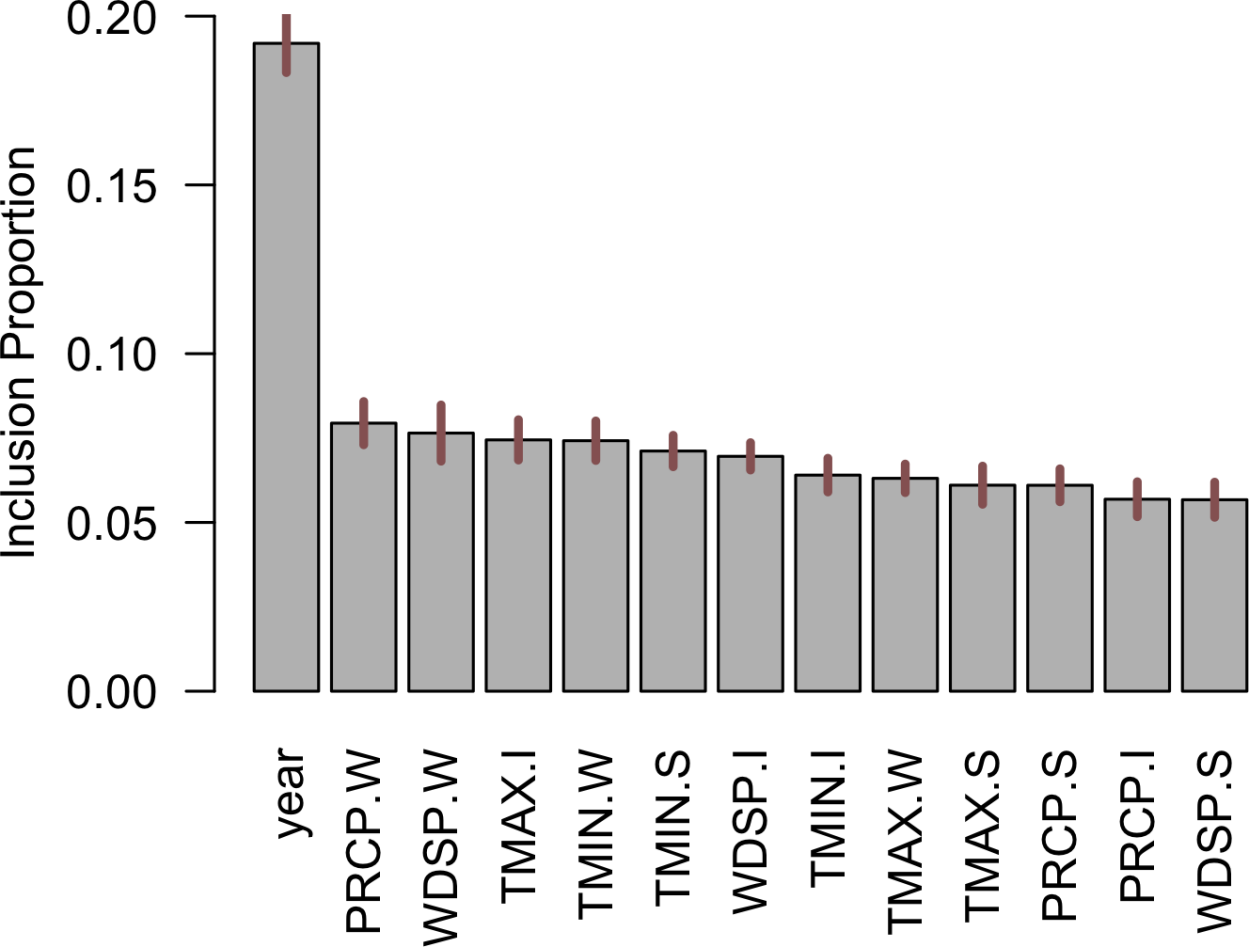
Ranking of variable importance in predicting end-use residential energy consumption.

The variable rankings in the commercial sector is different from that of the residential sector (Fig 11). For instance, unlike the residential sector, variable ‘year’ is identified as a considerably more important predictor of energy demand compared to all other climatic variables. This is as expected since a healthy and growing economy will boost commercial activities, thereby, increasing the demand. Moreover, in contrary to the residential sector, commercial buildings are often centrally owned and managed. Therefore, the climate sensitive portion of the commercial demand patterns is expected to be different from the residential sector’s, where the behavior is much more sensitive to the environmental conditions [11]. Out of the climate variables, it can be noticed that wind speed, precipitation levels and minimum temperatures during the winter months, together with temperatures during the summer and intermediate seasons rank as the most important predictors of the climate sensitive portion of the commercial energy demand.

**Fig 12:**
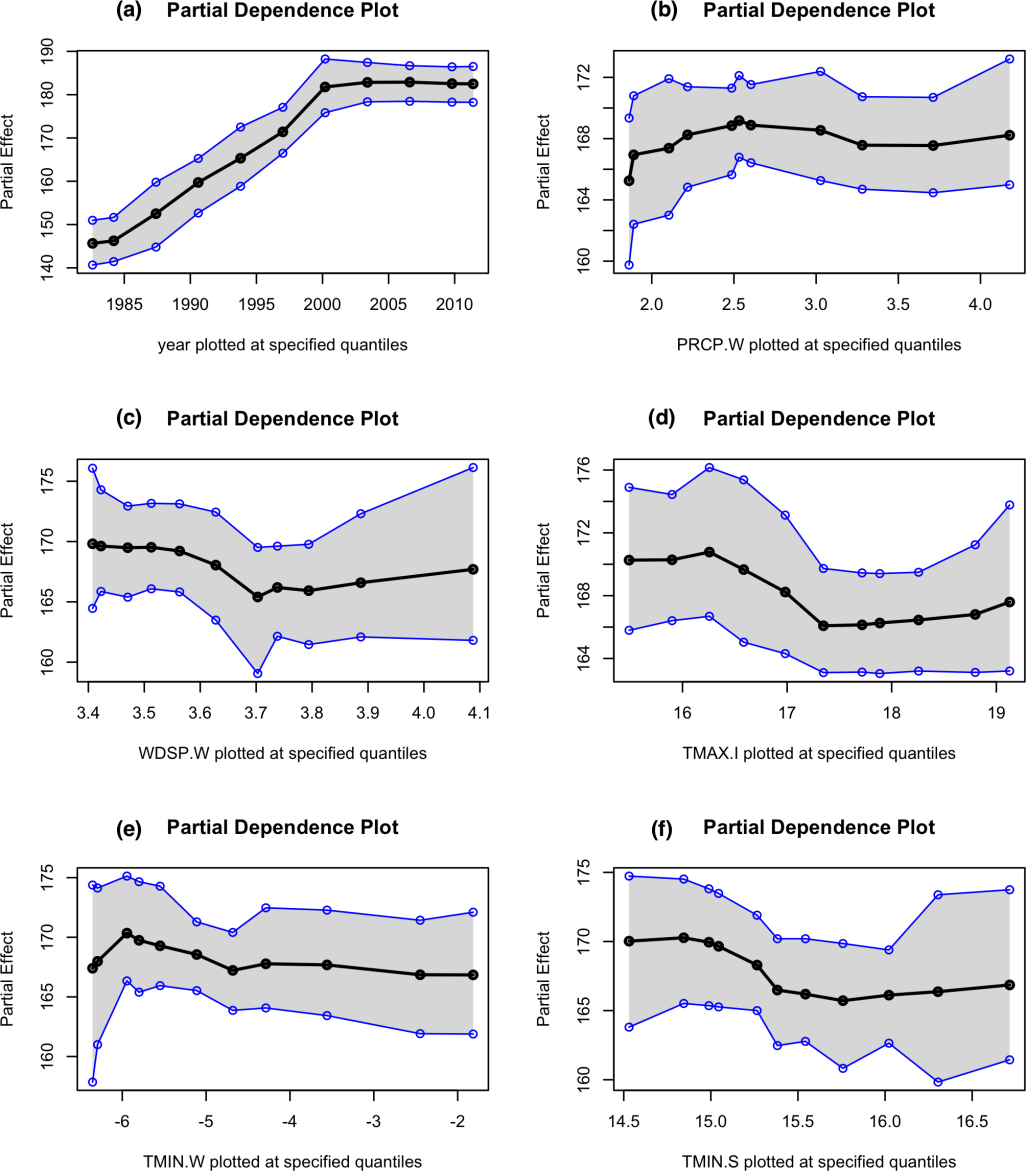
Partial dependencies of the top six key predictors: (a) year (b) precipitation during winter (in inches), (c) wind speeds during the winter season (in m/s), (d) maximum temperature (in °C) during the intermediate season, (e) minimum temperature the during winter (in °C), and (f) minimum temperature (in °C) during the summer, in the commercial sector.

The partial dependency plots of the top six predictors in the commercial sector is given in Fig 12. Like the residential sector, the variable ‘year’ is positively associated with energy demand until early 2000’s, after which the energy demand plateaus, largely due to the energy conservation and efficiency initiatives such as ‘Energizing Indiana’ that contained comprehensive energy-saving programs for offices and businesses in IN. Higher winter precipitations (Fig 12B) are associated with higher energy demand, since more energy is needed for space heating and water heating due to the precipitation-induced colder environments. Demand (for heating) will be higher when the wind speeds are higher than 3.7 meters per second (Fig 12C); accounting largely for the wind-chill effects during the months of December and January. Temperatures above 16°C (Fig 12D) during the intermediate seasons are associated with lower energy demand, as this indicates a comfortable temperature requiring lower levels of space heating or space cooling. Energy demand (for heating) is reduced as the minimum (negative) temperatures rise in the winter (Fig 12E). The minimum temperatures in the summer in the state of IN can still be quite low (in the range of 14–16°C) and higher (minimum) temperatures are also associated with reduced demand.

### RCP 4.5 and RCP 8.6 scenario-based projections

#### Aggregated energy projection

**Fig 13:**
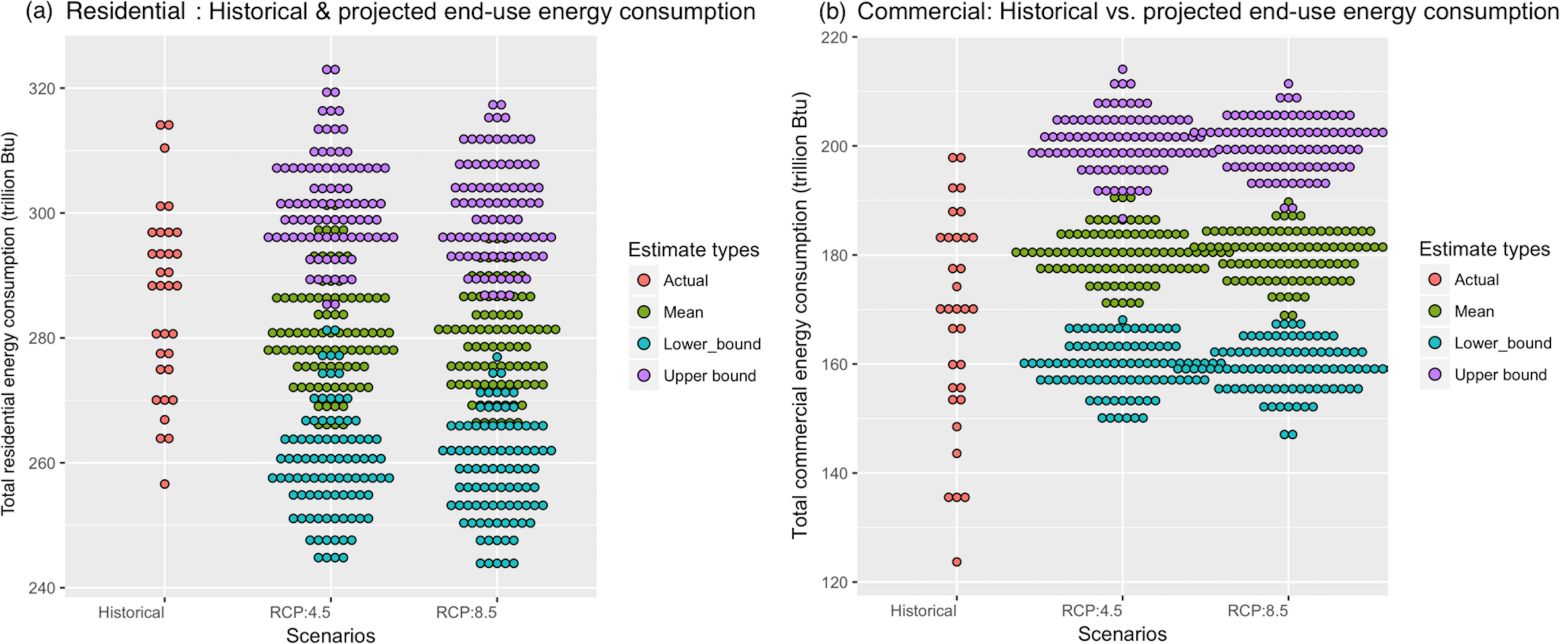
Dot-plot showing the scenario-based comparison of the total energy consumption for (a) the residential sector, and (b) the commercial sector.

The median values as well as upper and lower confidence bounds associated with the aggregated state-level energy demand in the residential and commercial sectors under the two scenarios of RCP 4.5 and RCP 8.5 are presented in Fig 13A and 13B. The first columns in the figures are associated with historical demand (1980–2013) and the second and third columns show the projections all the way to 2100 under RCP 4.5 and RCP 8.5 respectively. Under RCP 4.5, the median residential demand is projected to be 278.5 trillion Btu—a 3.5% decrease from the historical median (Table 1)—and can vary in the range of 243.7–323.2 trillion Btu. Under RCP 8.5 the median residential demand is projected to be 278.7 trillion Btu—a 3.5% decrease from the historical median—and could range between 243.0–276.9 trillion Btu. Fig 13A reveals that the future energy demand will be lower under the high emissions scenario (RCP 8.5) for the residential sector. This is not surprising since, in the residential sector in IN, the highest fraction of energy is used for space heating (historical demands ranging from 187.6–230.3 trillion Btu), followed by water heating (in the range of 64.7–79.4 trillion Btu). Least amount of energy is used for space cooling (historical demand ranging between 4.4–5.4 trillion Btu). Higher temperatures will, therefore, lead to reduced demand in the residential sector.

Unlike the residential sector, in the commercial sector, space cooling and space heating account for similar fractions of end-use energy demand; with the historical amounts ranging in 19.4–31.1 trillion Btu and 18.7–30.0 trillion Btu, respectively. The least amount of energy is consumed for water heating—historical demand ranging between 6.4–10.3 trillion Btu. It can be seen from Fig 13B that the demand in the commercial sector will be on-average substantially higher than the historical average values under both emission scenarios. More specifically, under the RCP 4.5 scenario, the average demand will be around 179.9 trillion Btu—a 5.1% increase than the historical median and it could range between 147.8–214.1 trillion Btu. Similarly, under the RCP 8.5 scenario, the projected median commercial demand will be 180.5 trillion Btu—a 5.4% increase from the historical median (Table 2) and could range between 146.4–211.4 trillion Btu under the uncertain future scenario.

#### End-use energy projections

As discussed in the Methodology section, we obtained the projections for the end-use energy demand by multiplying the projected aggregate sectoral energy demand by the generated probability distributions of space heating, space cooling and water heating fractions of the respective sectors. The estimated changes in these categories of end-use energy demands under different scenarios are shown in Tables 5 and 6. These final estimates can then be easily integrated in the existing energy-economy models as discussed before.

**Table 5:** Projected end-use demand (in Btu) for scape heating, space cooling and water heating under RCP 4.5 and RCP 8.5 in the residential sector (arranged in descending order of demands).

Scenario/Variable	Median	Minimum	Maximum	% change from historical median
RCP 4.5/ Space Heating	203.6	191.1	221.9	-3.5%
RCP 4.5/ Water Heating	70.19	65.86	76.49	-3.5%
RCP 4.5/ Space Cooling	4.74	4.44	5.16	-3.4%
RCP 8.5/ Space Heating	203.7	191.2	217.0	-3.5%
RCP 8.5/ Water Heating	70.23	65.90	74.79	-3.5%
RCP 8.5/ Space Cooling	4.74	4.45	5.04	-3.4%

**Table 6:** Projected end-use demand under RCP 4.5 and RCP 8.5 in the commercial sector (arranged in descending order of demands).

Scenario/Variable	Median	Minimum	Maximum	% change from historical median
RCP 4.5/ Space Cooling	28.24	26.66	30.04	5.1%
RCP 4.5/ Space Heating	27.16	25.64	28.89	5.1%
RCP 4.5/ Water Heating	9.35	8.82	9.95	5.0%
RCP 8.5/ Space Cooling	28.34	26.29	29.79	5.4%
RCP 8.5/ Space Heating	27.26	25.28	28.65	5.4%
RCP 8.5/ Water Heating	9.38	8.71	9.86	5.4%

It should be noted that the results summarized in Tables 5 and 6 are contingent on the assumption that the future distributions of end-use energy demand will resemble that of the past. However, in the presence of disruptive technologies that could shift the consumption patterns, our proposed framework is still applicable; in that case, the generated empirical distributions of the fractions of end-use consumptions will have to be updated. Moreover, we would like to highlight that in this paper, we projected the average end-use demand for a ‘statistically representative’ user (i.e., an *individual household* or an *individual commercial building*). The implication here is that our results characterize the climate sensitivity of end-use energy demands for an average ‘representative’ household in the state, which does not imply that all households will have similar energy demand curves. For instance, while based on our projected estimates, the average household heating energy in the year of 2050 will be around 200±15 Btu, the distribution of household heating energy load in Indiana could be as low as 20 Btu in a mobile home in rural Indiana to possibly up to 500 Btu in a mansion located in the wealthy Hamilton County. Moreover, it should also be stressed that since our estimates are reported on a per-household/building basis, our results are not sensitive to future population variations in the state. It is noteworthy that projected energy demand in the residential sector does not vary much under the two climate change scenarios. We hypothesize two major reasons for this outcome. First, due to the lack of availability of the statistically downscaled projection of the state-level mean dew point temperature, our model considered only the minimum and maximum temperatures which are inadequate indicators of the heat stress [11]. Moreover, our previous research showed that mean dew point temperature is a better predictor of the climate sensitive end-use demand as compared to the other climate variables [3,11]. Thus, with the inclusion of the mean dew point temperature in the model, we might be able to notice significant differences in the residential energy demand under the two climate change scenarios. Second, the projected wind speed data was not statistically downscaled as it showed statistically insignificant differences from the historical distributions. However, since wind speed was found to be an important predictor for the residential energy demand, availability of accurate wind speed projections might have an influence on the residential demand projections under the two scenarios.

## Conclusions

Reliable access to energy is critical for proper functioning of the modern society. Integrated resource adequacy planning in the energy sector hinges on the capability to accurately project the future trends in energy demand. However, projecting long-term trends in energy demand is an increasingly complex endeavor due to the uncertain emerging changes in climate, policy, regulatory environment, economy and technology among other factors. There exist many powerful energy-economy models that can be leveraged to project the long-term demand trends in the energy sector under various future scenarios related to technology and policy trends. However, majority of the existing energy-economy tools (e.g., MARKAL) are not able to exogenously account for climate variability and change. In this paper, we proposed a multi-paradigm framework to help extend the energy-economy models to be able to account for climate change. To illustrate the applicability of our proposed framework, we used the state of Indiana as a case study. We trained and rigorously validated a Bayesian, non-parametric predictive model with historical data on end-use energy demand and climatic conditions in Indiana to characterize the energy demand-climate nexus. We then used downscaled climate scenarios to project future demands under the two climate scenarios of RCP 4.5 and RCP 8.5. Finally, we harnessed detailed information on the energy used for space cooling, space heating and water heating in the residential and commercial sectors, to generate sampling distributions for these categories of end-use demand to transform the aggregate state-level estimates into individual household- and building-levels. The probabilistic estimates of end-use demand (with associated credible and prediction intervals) under each scenario could then be fed into MARKAL or other similar energy planning tools to facilitate accounting for climate variability and change.

Our analysis concluded that under both climate change scenarios of RCP 4.5 and RCP 8.5, the energy consumption for an ‘average’ household in IN is projected to decrease by 3.5% by 2100 due to less heating requirement during warmer winters. Moreover, by 2100, the energy demand for an ‘average’ commercial building is projected to increase by 5.1% and 5.4% under RCP 4.5 and 8.5, respectively (compared to the historical consumption over 1981–2013). The projected increase is due to the commercial sector’s greater use of cooling energy, relative to the residential sector, and higher projected daytime temperatures.

It should be noted that while our proposed framework accounts for demand variation due to climate change, it does not account for potentially higher intensity and frequency of climatic extremes (such as ice-storms and tornados) under climate change. Future work is needed to extend our paradigm to also account for such weather and climate extremes that could become much more intense and frequent under climate change, and have significant impacts on our energy sector in the future.
